# The Retinitis Pigmentosa Mutation c.3444+1G>A in *CNGB1* Results in Skipping of Exon 32

**DOI:** 10.1371/journal.pone.0008969

**Published:** 2010-01-29

**Authors:** Elvir Becirovic, Kostadinka Nakova, Verena Hammelmann, Roman Hennel, Martin Biel, Stylianos Michalakis

**Affiliations:** Munich Center for Integrated Protein Science CIPSM and Department of Pharmacy, Center for Drug Research, Ludwig-Maximilians-Universität, München, Germany; Ohio State University Medical Center, United States of America

## Abstract

Retinitis pigmentosa (RP) is a severe hereditary eye disorder characterized by progressive degeneration of photoreceptors and subsequent loss of vision. Two of the RP associated mutations were found in the CNGB1 gene that encodes the B subunit of the rod cyclic nucleotide-gated channel (CNGB1a). One of them (c.3444+1G>A) is located at the donor site of exon 32 and has been proposed to result in a frameshift and truncation of the last 28 aa of the corresponding protein. However, this ambiguous conclusion was not verified by experimental data. Recently, another study reported that the last 28 aa of CNGB1a harbor a motif required for the proper targeting of this subunit to rod photoreceptor outer segments. This suggests that defective targeting is the major cause for the RP phenotype in affected patients. Here, we investigated the splicing of c.3444+1G>A by exon trapping experiments and could demonstrate that instead of the proposed truncation of the last 28 aa this mutation leads to replacement of the last 170 aa of CNGB1a by 68 unrelated amino acids. The 170 aa deletion covers the complete distal C-terminus including the last 10 aa of an important alpha (αC) helix within the ligand-binding domain of CNGB1a. When expressed in a heterologous expression system the corresponding mutant full-length CNGB1a subunit was more susceptible to proteosomal degradation compared to the wild-type counterpart. In conclusion, our experimental data do not support the hypothesis proposed by the original study on the c.3444+1G>A mutation. Based on this, we suggest that apart from the defective targeting other mechanisms may be responsible for the RP phenotype in affected individuals.

## Introduction

Retinitis pigmentosa is a severe hereditary eye disorder characterized by progressive degeneration of photoreceptors and subsequent loss of vision. Two of the RP associated mutations were found in the *CNGB1* gene [1], [2] encoding the B subunit of the rod cyclic nucleotide-gated channel (CNGB1a). One of these mutations (c.3444+1G>A) is located at the donor site of exon 32. Although not clearly written in the original report, the conclusion can be drawn that c.3444+1G>A results in loss of the last 28 aa of CNGB1a. [2]. Recently, another study reported that the last 28 aa of CNGB1a harbour a motif required for the proper targeting of this subunit to rod photoreceptor outer segments [3]. This suggests the defective targeting to be the major cause for the RP phenotype in affected individuals.

However, our initial *in silico* analysis suggested that there is no possible splicing event by which the c.3444+1G>A mutation could lead to loss of only the last 28 aa of CNGB1a. This prompted us to analyze the effect of this mutation on splicing experimentally. Here, we demonstrate by means of exon trapping experiments that the c.3444+1G>A mutation on mRNA level results in skipping of exon 32 and, hence, to a frameshift after exon 31. Instead of truncation of the last 28 aa this frameshift on protein level leads to replacement of the last 170 aa of CNGB1a by 68 unrelated amino acids. When expressed in a heterologous expression system the corresponding mutant full-length CNGB1a subunit was more susceptible to proteasomal degradation compared to the wild-type counterpart. These results suggest that apart from the defective targeting other mechanisms may be responsible for the RP phenotype in patients affected by the c.3444+1G>A mutation.

## Results

We first performed *in silico* analysis in order to reconstruct the mechanism by which c.3444+1G>A could lead to truncation of the last 28 aa of CNGB1a. A plausible explanation would be the use of cryptic donor sites. Use of one potential cryptic donor site in exon 33 would indeed delete the sequence that encodes the last 28 aa. However, it would also lead to retention of intron 32. In this case, due to an intronic stop codon 171 bp after exon 32, the corresponding protein would lack all 95 aa encoded by exon 33.

**Figure 1:**
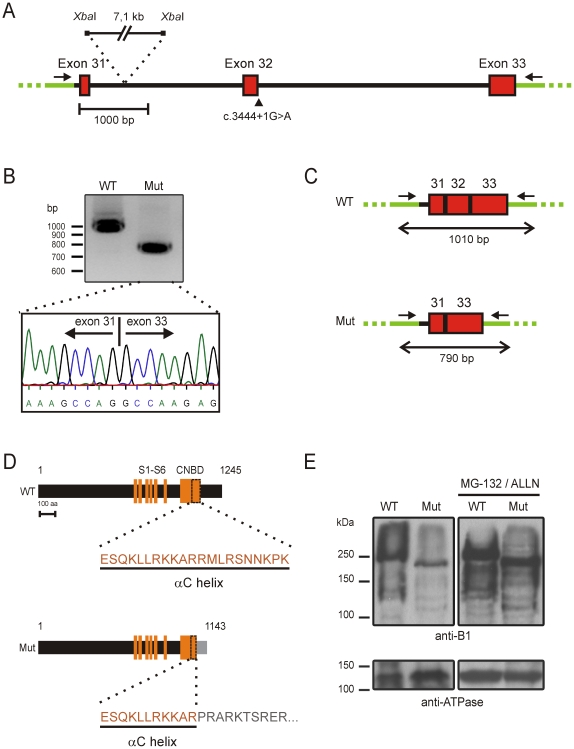
c.3444+1G>A mutation affects the splicing and expression of CNGB1. (A) Schematic representation of the minigene construct used for the exon trapping experiment showing the position of the c.3444+1G>A mutation (marked by an arrowhead) and the deleted intronic *Xba*I-fragment. Vector backbone sequence is depicted in green. (B) Revese transcriptase PCR from HEK293T cells transfected with mutant and wild type minigene constructs. The electropherogram for the c.3444+1G>A mutant shows the skipping of exon 32. (C) Scheme showing the splice products. The length of the respective PCR products is indicated by double arrows. (D) Schematic comparison of the WT and mutant protein demonstrating the lack of the entire distal C-terminus and the last 10 aa of the αC helix in the context of the c.3444G>A mutation. Skipping of exon 32 causes a frameshift which results in addition of 68 unrelated amino acids after aa position 1075 of the CNGB1a protein (highlighted in grey). The numbers represent the length of the respective proteins (1245 aa for WT and 1143 for the mutant). (E) Western blot of membranes isolated from HEK293T cells transfected with CNGA1 and wild type or mutant CNGB1a probed with anti-B1 (*top panel*) or anti-ATPase (*bottom panel*). The weaker expression of the mutant protein was normalized in the presence of the proteasome inhibitors MG-132 and ALLN. CNBD: cyclic nucleotide-binding domain. Primers are shown as arrows. S1–S6: transmembrane segments; WT: wild type, Mut: c.3444+1G>A mutation.

To investigate the impact of the c.3444+1G>A mutation experimentally, we first transfected HEK293T cells with wildtype and mutant minigene constructs designed to test splicing of exons 31–33 (see Materials and Methods and Fig. 1A). Sequencing of the PCR-amplified splicing products showed that the wild type construct was spliced correctly. In contrast, we found that on mRNA level the c.3444+1G>A mutation resulted in skipping of exon 32 (Fig. 1B) thereby leading to a frameshift after exon 31. As a result, the regular coding region of CNGB1a stops after amino acid 1075 followed by 68 unrelated amino acids. The deleted part of CNGB1a encompasses 170 aa and covers the complete distal C-terminus including the last 10 aa of the αC helix within the cyclic nucleotide-binding domain (CNBD) (Fig. 1C). To investigate the consequences of skipping of exon 32 on the full length protein we coexpressed the full-length mutant CNGB1a in HEK293T cells with the A subunit (CNGA1) that together with CNGB1a forms the native rod channel. In the western blot analysis using an antibody directed against the N-terminus of CNGB1a we could detect the expected 240 kDa band for the wild type CNGB1a. As anticipated, the mutant CNGB1a protein was smaller than the wild type counterpart. Furthermore, the expression level of the mutant CNGB1a was considerably reduced compared to the wild type CNGB1a (Fig. 1D, left). Since this difference in expression could be reversed by the addition of proteasome inhibitors, we concluded that the mutant protein is partially degraded by the proteasome (Fig. 1D, right).

## Discussion

In this study, we could verify the pathogenic effect of a previously reported splice site mutation in *CNGB1* experimentally. We were not able to reconstitute any splicing scenario that would lead to “a frameshift and truncation of the last 28 aa” of CNGB1a as suggested by the original study [2]. Using *in vitro* exon trapping experiments we could show that this mutation gives rise to skipping of exon 32. However, due to the limitation of exon trapping experiments we can not completely exclude the possibility that in photoreceptors the mutation may have other effects on splicing.

Based on our results obtained in HEK293T cells, we provide three possible mechanisms for the disease. (1) We found that expression of the mutant CNGB1a is compromised by the action of the proteasome. This may also be the case in rod photoreceptors resulting in loss of channel. Mutations that result in premature stop codons are known to trigger nonsense mediated mRNA decay (NMD) [4]. Since skipping of exon 32 gives rise to a premature stop codon, we can not exclude that c.3444+1G>A mutant transcripts are affected by NMD *in vivo*, which would also negatively affect channel expression. (2) Recently, it has been shown that the distal C-terminus of CNGB1a contains an ankyrin G binding motif responsible for the proper targeting of the channel to rod outer segments [3]. This domain is located within the deleted sequence in the mutant CNGB1a. Thus, if the channel is expressed, its targeting to rod outer segments may be affected by the mutation. (3) It has been shown that the structural integrity of the αC helix of the CNBD is crucial for proper channel gating [5], [6], [7]. Since the c.3444+1G>A mutation results in loss of the last 10 aa of the αC helix, the mutant channel, even if expressed at normal levels in rod outer segments, would be most probably non-functional. Which of these parameters (and to which extent) contributes to the disease in affected patients remains to be determined.

## Materials and Methods

### *In Silico* Splicing Analysis

*In silico* analysis was performed using the NNSplice 0.9 splice site prediction software (http://www.fruitfly.org/seq_tools/splice.html). The DNA sequence used for this analysis starts with exon 32 and ends with the stop codon of CNGB1 in exon 33.

### Exon Trapping Experiments

A DNA fragment starting from the last 55 bp of intron 30 and ending with the last 42 bp after the stop codon within exon 33 of *CNGB1* (Fig. 1A) was PCR amplified from human genomic DNA and sequenced. For cloning convenience, a 7.1 kb fragment of intron 31 flanked by *Xba*I sites was deleted. The final 6.4 kb minigene construct was subcloned into the pcDNA3 vector (Invitrogen). The c.3444+1G>A mutation was inserted using standard site directed mutagenesis. RNA was isolated from HEK293T cells transfected with wild type or mutant constructs. After cDNA synthesis (ThermoScript RT-PCR System, Invitrogen) and PCR amplification with vector specific primers the splicing products derived from the minigenes were sequenced.

### Heterologous Expression and Western Blot

Human full-length CNGA1 and CNGB1 channel subunits were PCR amplified from human retinal cDNA and subcloned into the pcDNA3 vector. Human total retinal RNA was kindly provided by Dr. M. Preising (University of Giessen). The full-length mutant CNGB1a cDNA was obtained by deleting the exon 32 of the full-length wild type CNGB1 cDNA. For western blotting experiments membranes were isolated from HEK293T cells transfected with CNGA1 and wild type or mutant CNGB1a as described previously [8]. The blot was probed with an antibody directed against the N-terminus of CNGB1a [9]. As loading control we used the anti-ATPase antibody (1∶1000, clone α6F, developed by D.M. Fambrough, obtained from the Developmental Studies Hybridoma Bank, Iowa) [10]. In proteasome inhibition experiments MG-132 and ALLN (25 µM each, Calbiochem) were added directly to the cells sixteen hours prior to harvesting.
